# Conversion of banana peel into diverse valuable metabolites using an autochthonous *Rhodotorula mucilaginosa* strain

**DOI:** 10.1186/s12934-022-01834-0

**Published:** 2022-05-28

**Authors:** Dagoberto Torres-Alvarez, Angel León-Buitimea, Alonso Albalate-Ramírez, Pasiano Rivas-García, Emanuel Hernández-Núñez, José Rubén Morones-Ramírez

**Affiliations:** 1 Facultad de Ciencias Químicas, Universidad Autónoma de Nuevo León, UANL, San Nicolás de los Garza, Nuevo León Mexico; 2 Facultad de Ciencias Químicas, Centro de Investigación en Biotecnología y Nanotecnología, Universidad Autónoma de Nuevo León, Parque de Investigación e Innovación Tecnológica, 66629 Apodaca, Nuevo León Mexico; 3 Grupo de Investigación en Bioprocesos Sustentables, Facultad de Ciencias Químicas, Centro de Investigación en Biotecnología y Nanotecnología, Universidad Autónoma de Nuevo León, Parque de Investigación e Innovación Tecnológica, km 10 Highway to International Airport Mariano Escobedo, Apodaca, Nuevo León Mexico; 4 Departamento de Recursos del Mar, Centro de Investigación y de Estudios Avanzados del Instituto Politécnico Nacional, Antigua carretera a Progreso km. 6, colonia Cordemex, 97310 Mérida, Yucatán Mexico; 5 CONACYT, Av. Insurgentes Sur 1582, Col. Crédito Constructor, C.P. 03940 Alcaldía Benito Juárez, Ciudad de Mexico Mexico

**Keywords:** *Rhodotorula mucilaginosa*, Banana peel, Carotenoids, Exopolysaccharide, Fatty acids

## Abstract

Low-cost substrates are an exciting alternative for bioprocesses; however, their complexity can affect microorganism metabolism with non-desirable outcomes. This work evaluated banana peel extract (BPE) as a growth medium compared to commercial Yeast-Malt (YM) broth in the native and non-conventional yeast *Rhodotorula mucilaginosa* UANL-001L. The production of carotenoids, fatty acids, and exopolysaccharides (EPS) was also analyzed. Biomass concentration (3.9 g/L) and growth rate (0.069 g/h) of *Rhodotorula mucilaginosa* UANL-001L were obtained at 200 g/L of BPE. Yields per gram of dry biomass for carotenoids (317 µg/g) and fatty acids (0.55 g/g) showed the best results in 150 g/L of BPE, while 298 µg/g and 0.46 mg/g, respectively, were obtained in the YM broth. The highest yield of EPS was observed in 50 g/L of BPE, a two-fold increase (160.1 mg/g) compared to the YM broth (76.3 mg/g). The fatty acid characterization showed that 100 g/L of BPE produced 400% more unsaturated compounds (e.g., oleic and ricinoleic acid) than the YM broth. Altogether, these results indicate that BPE is a suitable medium for producing high-value products with potential industrial applications.

## Introduction

There has been a growing interest from industry to use and produce non-contaminant or easily degradable chemicals [1]. One potentially alternative is designing and developing novel bioprocesses, which harness microbial synthesis to produce valuable metabolites [2, 3]. These metabolites are commonly high molecular weight molecules, such as biopolymers and active pharmaceutical compounds, which cannot be easily manufactured by chemical synthesis. The production processes of these compounds typically create polluting sub-products, such as the synthesis of oil-based fuels [2–6]. Almost any microorganism can be used for microbial synthesis; however, not all can produce valuable and complex molecules as part of their natural metabolism. Among the different microorganisms to use in bioprocesses, yeasts have great potential due to a complex and variable metabolism that make them optimal to produce a vast number and diversity of molecules [7]. Moreover, yeasts offer many advantages, such as fast growth (3–7 days), high tolerance to adverse culture conditions (e.g., temperature, pH, and oxygen concentration) [8–10], and they can easily be genetically manipulated [11–13].

The genre *Rhodotorula* includes budding yeasts with a complex metabolism that allows the production of a wide array of valuable metabolites in chemical, pharmaceutical, and fuel industries [7, 14, 15]. Moreover, this genre offers the ability to use different carbon sources [16–20]. One of the most studied species in this genre is *Rhodotorula mucilaginosa* (*R*. *mucilaginosa*) due to its capacity to grow in harsh conditions such as highly contaminated environments, conditions of high salinity [21], low pH soils [22], rivers with a high concentration of heavy metals [23, 24] and shallow temperatures (below 5 °C) [25]. Also, *R. mucilaginosa* can use complex sugars as a precursor of their central metabolism, unlike other microorganisms (like bacteria) that can only use simple sugars such as glucose or fructose [26–28]. This characteristic allows the use of complex growth media as nutrient sources for cell division and the production of desired metabolites, being waste products the best alternative to fuel microbial synthesis and decrease production costs.

A wide array of waste components, such as molasses, food industry effluents, and milk serum, have been studied to use as a broth to grow different species of *Rhodotorula*. The results showed that the growth was similar to that observed in commercial growth media, such as Yeast Malt (YM) or Yeast peptone dextrose (YPD) [19, 29]. Fruit peel waste is the unused or unconsumed parts of a fruit. They are abundantly available worldwide and are a rich source of bioactive compounds. Fruit peels still contain macronutrients (carbohydrates and proteins) and phytochemicals (phytosterols, phenols, and catechol derivatives) [30, 31]. Therefore, fruit peel wastes have received significant attention due to their potential applications as fertilizers, chemical synthesis of active carbon, adsorption of heavy metals or dyes, and nutrient sources to grow microorganisms [32–34].

Banana contains many carbohydrates in the pulp. However, amino acids and essential minerals (e.g., phosphorus, calcium, and potassium) are much higher in the peel than in the banana pulp [35, 36]. Moreover, starch and free sugars represent around 35% of the whole fruit's dry weight, while lignocellulosic biomass represents 25% [37]. Also, most of the compounds can be obtained by hot water extraction, and the substrates can act as nutrient sources to support microbial growth [38]. Therefore, banana peel extract (BPE) as a growth medium represents a cheap and inexpensive suitable alternative for obtaining high-value products and helps to solve the severe environmental pollution problem caused by the extensive disposal of nutrient-rich banana peels into natural habitats [39, 40]. This study evaluates using a low-cost growth medium such as BPE to cultivate *R. mucilaginosa* UANL-001L. Moreover, carotenoid, exopolysaccharide (EPS), and lipid production are quantified and compared to those obtained when *R. mucilaginosa* UANL-001L is cultivated in a commercial YM medium. The results in this work shine light on how the carbon source influences cell metabolism and the production of valuable compounds in *R. mucilaginosa* UANL-001L.

## Material and methods

### BPE preparation

Banana peels from the Cavendish variety (AAA) were obtained from a local market in Monterrey, Mexico. Then, 50, 100, 150, 200, and 300 g of cut peels were put into a beaker with 1 L of deionized water and heated until boiling (80 °C) by a heating plate. After 5 min of boiling, the beaker was taken off the plate and cooled down at room temperature for 15 min. A prefiltration was made using gauze to trap the big pieces of the peel. Afterward, filtration was carried out twice in a Buchner vacuum filter flask with a 2-micron pore size filter (Whatman). The filtered extract was divided into three 500 mL Erlenmeyer flasks with 300 mL each and sterilized by autoclaving [16].

### BPE characterization

Solids profile was obtained using gravimetric technics. A 5 mL extract sample was placed in a ceramic crucible, dried at 105 °C, and weighed to obtain the total solid mass. Moisture content was determined by subtracting the weight of the sample before and after the drying process. The dried sample was heated at 550 °C inside a furnace to volatilize all the organic matter, and then the ashes were weighed. Mass of volatilized solids was obtained by subtracting the ashes' weight from the dry biomass [31, 37].

Nitrogen content was determined using the Kjeldahl total nitrogen method [41]. Briefly, a 0.1 g dried sample was mixed with reactive selenium and H_2_SO_4_ at 500 °C for 30 min. The produced (NH_4_)SO_4_ was distilled and recovered in a boric acid 4% solution (pH: 4.65). Nitrogen concentration was established by quantification of the NH_4_^+^ ions in the solution.

### Microbial culture

The yeast strain used in this work was *Rhodotorula mucilaginosa* UANL-001L (*R. mucilaginosa* UANL-001L). This native yeast was previously isolated from the Pesqueria river’s waters in the Northeast of Mexico (State of Nuevo Leon) [23]. Dry Yeast Malt growth media (YM) (Difco) was prepared according to the manufacturer’s instructions. An overnight culture of *R. mucilaginosa* UANL-001L was grown in a YM medium at 28 °C and 300 rpm. Then, BPE and YM cultures were inoculated with one milliliter of the overnight culture to an initial OD_600_ of 0.1. The cultures were grown for 4–6 days as described above. One milliliter of the sample was taken every 24 h to record the OD_600_ sample using a spectrometer (Optizen 2120 UV Plus). All experiments were carried out in triplicates (n = 3).

### Determination of reducing sugars

Quantification of the reducing sugars in the *R. mucilaginosa* UANL 001L BPE and YM growth media was performed through the dinitrosalicylic acid (DNS) method [42]. A calibration curve (0.1 to 10 g/L glucose) was used to calculate the samples’ sugar concentration. Briefly, 10 mL of each sample were taken, centrifugated at 4200×*g* for 10 min, and the supernatant was separated from the biomass. One milliliter (1 mL) of supernatant was dispensed in a test tube, and two milliliters (2 mL) of the DNS reagent were added. The mixture was heated at 90 °C for 5 min. The absorbance was measured with a UV–Vis spectrophotometer at 540 nm, using a blank as a control. Reducing sugars were quantified at 0, 24, 48, 72, 96, and 144 h of the growth of *R. mucilaginosa* UANL 001L in BPE and YM media. For BPE, (NH_4_)_2_SO_4_:1M was added in a 1:5 (ammonia sulfate: reducing sugars) ratio as a nitrogen and sulfur source. Experiments were performed in triplicates (n = 3).

### Extraction and determination of total carotenoids

After 5 days of growth, the extraction of carotenoids from *R. mucilaginosa* UANL 001L growth medium (BPE and YM) was carried out. 0.5 g freeze-dried biomass sample was mixed with 10 mL of acetone and ultrasonicated at 40 kHz with glass beads for 10 min. Then, the samples were centrifugated at 7400×*g* for 5 min, and the supernatant was collected. This process was repeated to make sure; the pigment extraction was complete. The samples were concentrated in 2 mL using a vacuum concentrator (Savant SPD 2010 SpeedVac, Thermo Scientific), and the OD_480_ was recorded. All experiments were carried out in triplicates (n = 3). Total carotenoids were obtained applying the Deming et al. [43] equation:

$$ Carotenoids\;concentration \left( {\frac{{ Carotenoids\;weight \left( {{\text{mg}}} \right)}}{{Growth media\;volume \left( {\text{L}} \right)}}} \right) = \frac{1000 ADV}{{0.16 W}} $$

$$ Carotenoids\;content \left( {\frac{{ Carotenoids\;weight \left( {\upmu {\text{g}}} \right)}}{{Yeast\;dry\;biomass \left( {\text{g}} \right)}}} \right) = \frac{1000 ADV}{{0.16 W}}, $$

A is the absorbance at 480 nm, D is the dilution ratio, 0.16 = extinction coefficient of carotenoids, V is the volume of acetone, and W is the weight of dried biomass (g).

### Extraction of EPS

After 5 days of *R. mucilaginosa* UANL 001L growing in BPE and YM media, the supernatant was collected by centrifugation at 7400×*g* for 10 min. EPS extraction was made following the Vazquez-Rodriguez [44] method. Briefly, the supernatant was filtered using a 2 µm and then a 0.2 µm pore size filter. The supernatant was separated into 50 mL falcon tubes containing 10 mL of supernatant and 30 mL of 95% ethanol to precipitate the EPS. The samples were stored at − 20 °C for 12 h, and then the mixture was centrifuged at 16,700×*g* for 15 min. The precipitated EPS was washed twice with 70% ethanol to eliminate molecules attached to the EPS (such as proteins, peptides, or cell debris) [45]. Each assay was performed in triplicates. The resulting EPS was freeze-dried lyophilized for 24 h (Labconco Freezone-6 model), and the yield was calculated.

$$ EPS\;concentration = \frac{{Exopolysaccharide\;weight \left( {{\text{mg}}} \right)}}{{Growth\;media\;volume \left( {\text{L}} \right)}} $$

$$ EPS\;content = \frac{{Exopolysaccharide\;weight \left( {{\text{mg}}} \right)}}{{Yeast\;dry\;biomass\;weight \left( {\text{g}} \right)}} . $$

### Extraction of total lipid content and identification of fatty acids

The Folch method [46] was carried out to perform the lipid extraction. After 6 days of yeast growth in BPE and YM, the biomass was separated from the supernatant by centrifuging at 7400×*g* for 10 min. The biomass was freeze-dried and stored at 80 °C. Four grams of biomass were homogenized in 12 mL of 2:1 chloroform: methanol (v/v) mixture, and then 4 mL of 10% NaCl was added. After 10 min, the upper phase was discarded, and the bottom phase was extracted and washed using 70% ethanol in a Büchi rotavapor R-100. The lipid extract was stored at − 80 °C for further analysis. Each assay was performed in triplicates.

$$ Fatty\;acids\;concentration = \frac{{Fatty\;acids\;weight \left( {\text{g}} \right)}}{{Growth\;media\;volume \left( {\text{L}} \right)}} $$

$$ Fatty\;acids\;content = \frac{{Fatty\;acids \left( {{\text{mg}}} \right)}}{{Yeast\;dry\;biomass\;weight \left( {\text{g}} \right)}} . $$

### Fatty acids characterization

The extract fraction was analyzed on a Trace GC Ultra gas chromatograph coupled to an ion trap mass spectrometer (GC–MS) ITQ 900 (Thermo Fisher Scientific, Waltham, MA). An Agilent HP-5MS fused silica column (30 m 9250 μm 90 25 μm) was used. The mass spectrometer detector operated in mass scanning mode was used for quantification. The chromatographic conditions were as follows: carrier gas, helium (1 0.1 mL/min); injection mode, splitless; injector and detector temperatures, 270 and 250 °C, respectively. The following program was used to analyze specific fatty acids: 70 °C for 35 min; ramp at 10 °C min 1 to 300 °C, and hold 5 min.

### Statistical analysis

Experiments were conducted in triplicates; values are presented as mean (n = 3) ± standard deviation. The same letter represents no significant differences (*p* < 0.05). Analysis of variance (ANOVA) followed by Tukey’s *post-hoc* test was performed to identify statistically significant differences between different treatments.

## Results and discussion

### Profile of total solids and nitrogen (N) content in BPE

Components in BPE were determined by gravimetry and the Kjeldahl method. The results are summarized in Table 1. Our data indicated that the moisture content of banana peels varied from 89.66 to 90.14%, and the total solids represented about 9.84 ± 0.70%. Volatile solids of the banana peel were about 87.71 ± 1.36% of the dry biomass, in which carbon and nitrogen-based molecules can be found. The ash content ranged from 11.87 to 12.48%, indicating the presence of metals, but mostly, alkali metals such as potassium and calcium [31, 37]. The total Kjeldahl nitrogen was found to vary in the range of 2.06 to 2.74 g/L. The compositional analysis results performed on banana peels were similar to those reported by other authors [47–49].

**Table 1:** Results of compositional analysis performed on BPE

Parameter	Sample 1	Sample 2	Sample 3
Moisture (%)	89.79 ± 0.08	89.66 ± 1.14	90.14 ± 0.9
Total solids (%)	10.21 ± 0.08	10.34 ± 1.14	8.97 ± 0.9
Volatile solids (%db)	88.13 ± 0.91	87.49 ± 1.65	87.52 ± 1.53
Ash (%db)	11.87 ± 0.91	12.51 ± 1.65	12.48 ± 1.53
Total nitrogen (%db)	2.66 ± 0.002	2.65 ± 0.002	2.63 ± 0.0
Total nitrogen (g/L)	2.71 ± 0.002	2.74 ± 0.002	2.06 ± 0.0

*db* dry biomass

### Kinetic of *R. mucilaginosa* UANL 001L growth in YM medium and BPE

**Fig. 1:**
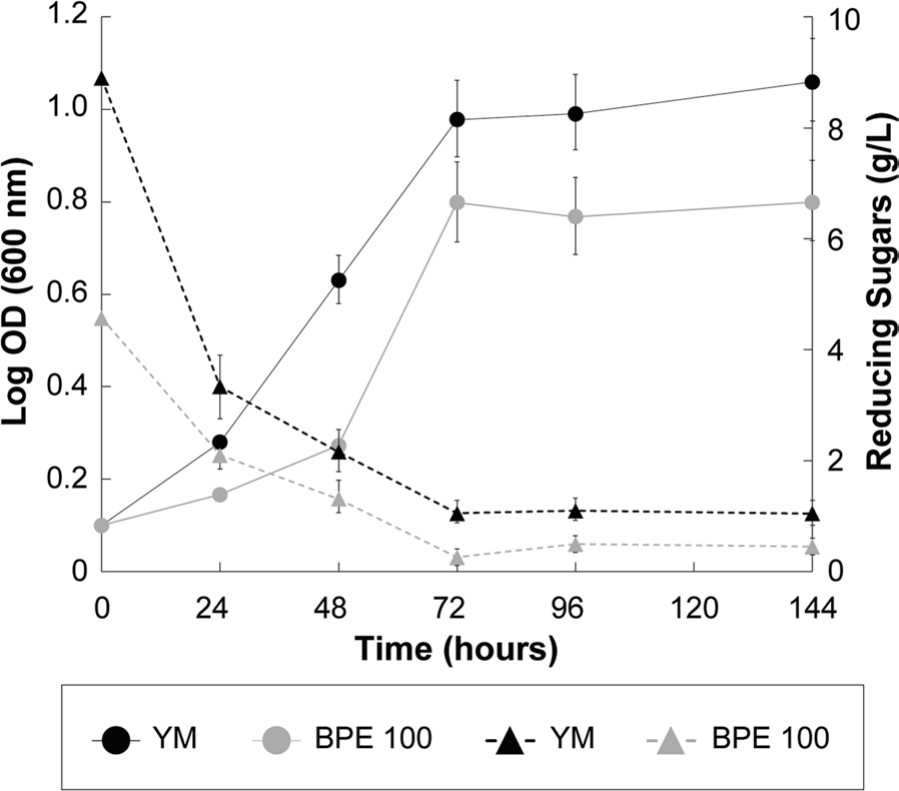
Growth kinetics and reducing sugar consumption of *Rhodotorula mucilaginosa* UANL 001L in YM broth and 100 g/L BPE medium. (Circles correspond to growth OD, and triangles correspond to the concentration of reducing sugars). Each point represents the mean ± SD, n = 3 a p-value < 0.05 was statistically significant (*) between treatments and control

The growth of *R. mucilaginosa* UANL 001L was first evaluated in YM broth, an optimal commercial medium. Exponential growth was observed for the first 72 h until yeast entered the stationary phase (Fig. 1). The maximum absorbance (Log OD_600_) observed in the growth curve was 1.06, which correlates with the 5.3 g/L obtained from dry biomass (Table 2). Some reports have measured dry biomass in different *R. mucilaginosa* strains and have reported from 6.5 to 8 g/L after 5–10 days of growth at 22–28 °C [29, 50]. Sugar consumption was also monitored during yeast growth. Our results showed that *R. mucilaginosa* UANL 001L consumed glucose as a carbon source during the first 72 h (exponential growth) (Fig. 1). The differences in the maximal growth and dry biomass among the present work and previous reports may be related to the aeration rate and/or strain genetic variability.

**Table 2:** Growth of *Rhodotorula mucilaginosa* UANL 001L in different BPE concentrations compared with YM broth

Sample	Biomass concentration		Growth rate (h^−1^)	Duplication time (h)	Reducing sugars consumption (g/L)
	OD	Biomass g/L			
YM	11.47 ± 0.392^a^	5.3 ± 0.187^a^	0.069^a^	10.05 ± 0.082^a^	7.08 ± 0.37^a^
BPE (g/L)					
50	5.44 ± 0.26^c^	2.7 ± 0.131^c^	0.052^c^	13.43 ± 0.019^c^	1.69 ± 0.028^c^
100	6.82 ± 0.281^b^	3.6 ± 0.147^b^	0.059^b^	11.74 ± 0.05^b^	4.01 ± 0.022^b^
150	6.87 ± 0.227^b^	3.3 ± 0.117^b^	0.066^a^	10.44 ± 0.074^a^	5.79 ± 0.109^b^
200	7.32 ± 0.202^b^	3.9 ± 0.108^b^	0.069^a^	10.11 ± 0.071^a^	7.75 ± 0.167^a^

Each point represents the mean ± SD, n = 3. The same letter represents no significant difference (p < 0.05)

The growth of *R. mucilaginosa* UANL 001L was evaluated in 100 g/L BPE following the previously described conditions for the YM broth. Our results showed that *R. mucilaginosa* UANL 001L growth was slightly slower than in YM broth within the first 48 h; however, the maximum peak was reached simultaneously (72 h) even though the maximum yield observed is 32% lower for the growth in BPE medium than the growth in YM broth (Fig. 1). This effect can be attributed to an adaptation period of *R. mucilaginosa* to different growth conditions. The maximum biomass concentration was observed at 72 h, reaching an absorbance of 0.833 log OD_600_, which correlates with 3.6 g/L of dry biomass (Table 2). Consumption of reducing sugars was observed during the first 72 h; then, no significant differences were observed up to 144 h of growth. These results suggest that reducing sugars (e.g., monosaccharides and disaccharides) found in the BPE were consumed as a carbon source for *R. mucilaginosa* growth (Fig. 1).

It has been reported that glucose and fructose are the main two significant sugars reported in the banana peel [31, 36]. In contrast, due to low solubility in hot water, starch was not expected to be present in the extract. At the same time, sucrose concentration is minimal compared to the other monosaccharides (glucose and fructose) since its hydrolysis occurs at high temperatures (e.g., autoclaving) [51]. Many reports have demonstrated that different *Rhodotorula* strains are able to intake simple sugars and grow in different types of media, such as those rich in molasse and waste effluents [19, 20, 52]. The two major reducing sugars (glucose and fructose) have been reported in very similar concentrations in banana peels [37]. Thus, our results suggest that *Rhodotorula mucilaginosa* UANL 001L can use the available monosaccharides in BPE for its cell growth.

### Growth curve of *R. mucilaginosa* UANL 001L using different BPE concentrations

The growth of *R. mucilaginosa* UANL 001L was evaluated in different concentrations of BPE and compared to YM broth after 7 days of culture. The results are presented in Table 2. As expected, the maximum biomass concentration was observed in YM broth, with an optical density (OD_600_) of 11.47, which corresponds to 5.3 g/L of dry weight biomass. Regarding the yeast growth in different concentrations of BPE, no significant differences were observed between 100, 150, and 200 g/L, and the biomass concentration ranged from 3.3 to 3.9 g/L. On the other hand, 50 g/L BPE showed the lowest biomass concentration with 2.7 g/L. Regarding the growth rate and duplication time of *R. mucilaginosa* UANL 001L, it can be observed that 50 and 100 g/L BPE exhibited the lowest growth rates (0.052 and 0.059 g/h) and, therefore, the highest duplication times (13.43 and 11.74 h^−1^); interestingly, 150 and 200 g/L BPE had similar results to those observed in YM broth. Regarding the quantification of reducing sugars, the reducing sugar consumption increased in a concentration-dependent manner after increasing doses of BPE. It can be observed that as BPE concentration increases (50, 100, and 150 g/L), the consumption of reducing sugars also increases (1.69, 4.01, and 5.79 g/L, respectively). Interestingly, at 200 g/L BPE, the consumption was even higher than YM broth (7.08 vs. 7.75 g/L, respectively). Thus, our results show that BPE seems to be an optimal growth media for *R. mucilaginosa* UANL 001L since it can use carbon and nitrogen sources present in the BPE to grow.

### Evaluation of culture media to produce valuable secondary metabolites

**Fig. 2:**
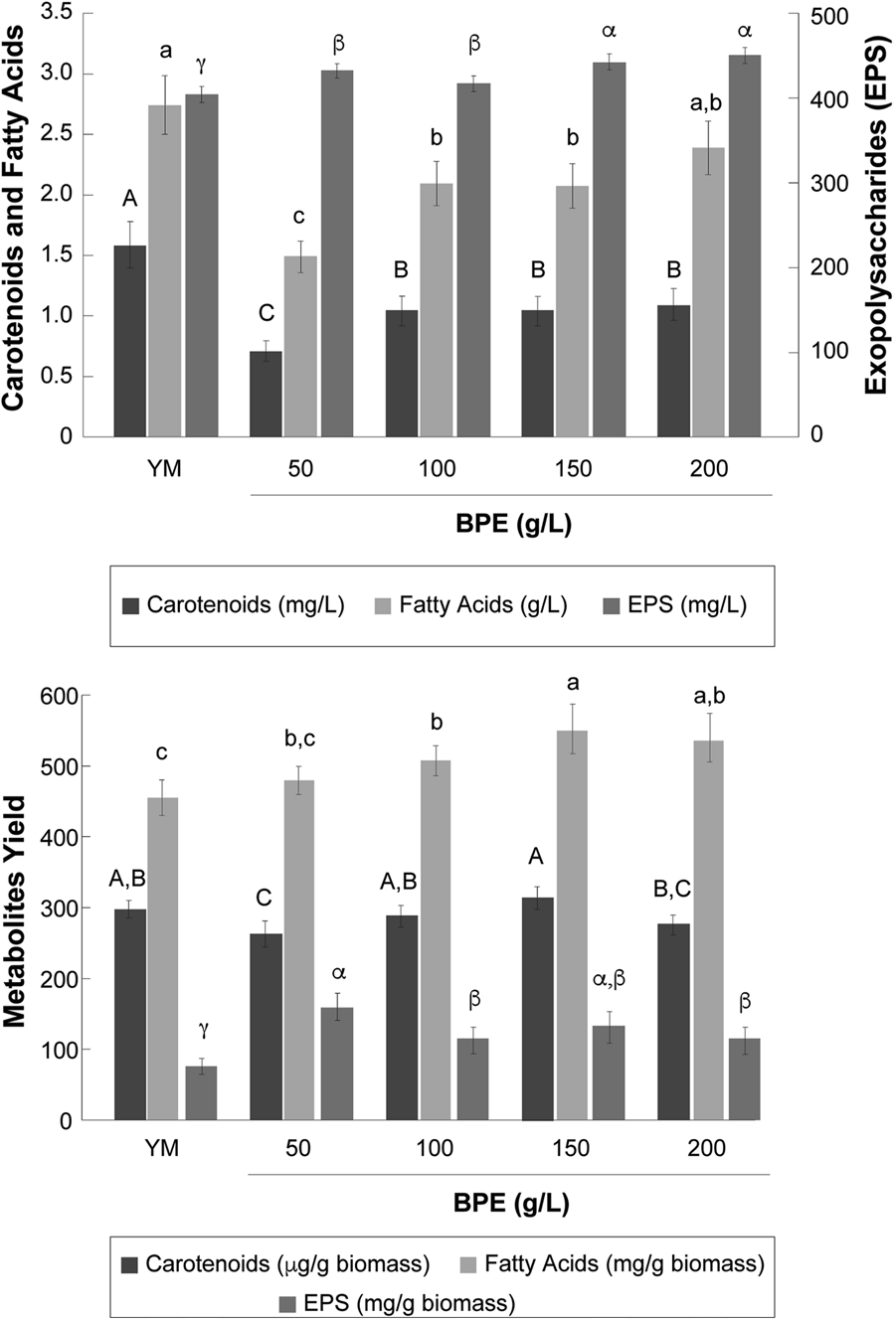
Production of carotenoids, exopolysaccharide (EPS), and fatty acids by *Rhodotorula mucilaginosa* UANL 001L in YM broth and BPE after 5 days of growth: **a** Concentration (mg/L or g/L), **b** Content (mg product/g biomass or mg product/g biomass). Each bar represents the mean ± SD, n = 3. The same letters did not differ significantly at p < 0.05

After 5 days of culture, the production of secondary metabolites (carotenoids, EPS, and fatty acids) by *R. mucilaginosa* UANL 001L was compared between BPE and YM broth (Fig. 2). Carotenoids are one of the most common valuable metabolites produced by *Rhodotorula* species. From the results obtained for the dry mass of yeast, we calculated the yield of carotenoid produced per gram of dry biomass (Fig. 2). Our results showed that *R. mucilaginosa* UANL 001L produced 5.3 g/L dry biomass while the carotenoid yield was 298 ± 12 µg/g of dry biomass in the YM broth. No significant differences were observed in the carotenoid yield between 100, 150, and 200 g/L BPE (291 ± 25, 317 ± 16, and 279 ± 24 µg/g of dry biomass, respectively) (Fig. 2). Thus, our results show that carotenoid production may be similar when BPE and YM are used as growth media. Carotenoids yield in different *Rhodotorula mucilaginosa* strains are presented in Table 3.

**Table 3:** Carotenoids yield per dry biomass in different *R. mucilaginosa* strains

Microorganism	Substrate^a^	Substrate concentration (g/L)	Carotenoid yield (µg/g)	Additional details	References
*R. mucilaginosa* UANL001	Banana peel extract	50	268 ± 19		This study
		100	291 ± 25		
		150	317 ± 16		
		200	279 ± 24		
*R. mucilaginosa* CCY 20-7-31	Glucose	40	62.50 ± 28.20	Potato extract with 5% salt (salt stress)	[55]
	Glucose + whey	40 + 7	422.30 ± 14.88		
	Glucose + potato extract	40 + 7	1535.60 ± 156.52		
*R. mucilaginosa* ATCC 66034	YPD + potato wastewater + Glycerol	Glycerol: 30	110	Glycerol above 100 g/L cause inhibition	[54]
		Glycerol: 50	110		
*R. mucilaginosa*	Alperujo water	100%	800	Alperujo water as only nutrients source	[68]
	Alperujo extract	30	700		
R. *mucilaginosa* ATCC 66034	Potato wastewater + glycerol	Glycerol: 30	88 ± 9	Cultured at 20 °C	[54]
R. *mucilaginosa* NRRL-2502	Molasses sucrose	20	21,200		[19]
	Whey lactose	13.2	35,000		
R. *mucilaginosa* MTCC-1403	Onion peel + mung bean husk	Sugar content 70.7	710.33 ± 27.87	Aqueos extract	[69]
	Potato skin + potato pods	Sugar content 30.8	538.81 ± 18.36		
	Onion peel + mung bean husk	Sugar content 79.99	717.82 ± 27.64	Acidic extract	
	Potato skin + potato pods	Sugar content 62.1	545.13 ± 28.77		
R. *mucilaginosa* WEP	Waste extract	Sugar content 14	1250	C/N ratio adjusted to 20:1 in waste extract	[70]
	Rice straw	Sugar content 5	441.17		

*YPD* yeast extract–peptone–dextrose medium

^a^Main carbon source

Similar results have been reported in different genera of *Rhodotorula*, as in the case of *Rhodotorula acheniorum* and *Rhodotorula glutinis*. Nasrabadi and Razavi carried out the optimization of β-carotene production by a mutant of the lactose-positive yeast (*Rhodotorula acheniorum*) from whey ultrafiltrate. The results showed that it was possible to achieve the highest level of β-carotene (262.12 ± 1.01 mg/L) [53]. Kot et al. showed that *R. gracilis grew* in media containing potato wastewater and glycerol and increased the biosynthesis of lipids and carotenoids (360.4 µg/g dry weight) in the presence of osmotic stress and low-temperature [54].

The production properties of several carotenogenic yeasts were evaluated by Marova et al*.* The highest yields were obtained in *R. glutinis* CCY 20-2-26 cells cultivated on whey medium (45 g/L of biomass enriched by 46 mg/L of beta-carotene) and in *R. mucilaginosa* CCY 20-7-31 grown on potato medium and 5% salt (30 g/L of biomass enriched by 56 mg/L of beta-carotene) [55]. Moreover, Schneider et al. investigated the production of microbial lipids for biodiesel production and high-value carotenoids by *Rhodotorula glutinis* combined with the use of brewery wastewater as a carbon source. Total average carotenoid contents ranged between 0.6 and 1.2 mg/L and with high proportions of β-carotene (∼ 50%) in the wastewater treatments [52, 53]. In our growth conditions, *R. mucilaginosa* UANL 001L seems to be an acceptable producer of carotenoids; nevertheless, results from other studies suggest that different nutrients [e.g., waste materials (whey, potato)] led to increased carotenoid production. Therefore, the continuation of this study should emphasize further substrate development since the incorporation of cheap waste substrates could improve carotenoid production in the BPE medium.

Furthermore, factors such as nitrogen sources have been explored to improve cell growth and carotenoid yield. For example, it has been described that an organic source like asparagine may improve the production of pigments compared to inorganic ammonia salts, notwithstanding it can significantly increase the production total cost [56]. Finally, genetic engineering could also be pointed out as an alternative. The effect of the overexpression of the carotenoid-related gene HMG1 has been evaluated with a small but positive outcome [57]. Therefore, more studies with different and multiple enzyme modifications should be conducted to demonstrate the effectiveness of this strategy.

Microbial EPS have been applied in the food, pharmaceutical, and biomedical industries. Previous reports have demonstrated that EPS has antimicrobial activity against bacterial pathogens [44]. Therefore, in the present work, we use BPE as growth media to explore optimal concentration to increase EPS biosynthesis in *R. mucilaginosa* UANL-001L.

First, EPS biosynthesis in *R. mucilaginosa* UANL-001L was studied in YM broth. Our results showed an EPS production of 404.5 ± 16.2 mg/L after 5 days of culture. Second, the production of EPS in *R. mucilaginosa* UANL-001L was 432.4 ± 68.3, 417.4 ± 27.1, 442.2 ± 44.2, and 451 ± 38.8 mg/L in 50, 100, 150, and 200 g/L of BPE media, respectively. No significant differences were observed between BPE concentrations. However, a slightly increased EPS production (3–12% higher) was observed in BPE compared to YM broth.

Numerous studies have reported EPS production by several bacteria, algae, fungi, and yeasts. EPS produced by *Candida* strains reached 0.4 to 2.8 g/L, while *R. minuta* strains and *Cryptococcus flavus* have produced 1.94–2.62 g/L and 2.7 to 3.6 g/L of EPS, respectively [58, 59]. It has also demonstrated that different growth conditions can significantly improve EPS production. Ghada et al. [45] evaluated the effect of pH, temperature, and different nutrients concentrations on EPS yield in *R. glutinis*, showing that, in optimal conditions, EPS production increased from 0.4 to 2.6 g/L. Moreover, EPS production in different *Rhodothorula mucilaginosa* strains has been reported (Table 4). In these studies, glucose, peptone/glycerol, or glucose/yeast extract have been used as carbon sources with EPS production ranging from 3.32 to 28.5 g/L [60–64].

**Table 4:** EPS concentration in different *R. mucilaginosa* strains

Microorganism	Growth media	Carbon source (g/L)	EPS concentration (g/L)	Potential application	References
*R. mucilaginosa* UANL001	Banana peel extract	50	0.432 ± 0.019	Antimicrobial	This study
		100	0.417 ± 0.023		
		150	0.443 ± 0.018		
		200	0.45 ± 0.021		
*R. mucilaginosa* CICC 33013	YPD medium	Glucose: 20	6.2	Antioxidation and anti-carcinoma activity	[60]
*R. mucilaginosa* Rho	PDB medium	Glucose: 20	3.328	Removal of heavy metals	[61]
*R. mucilaginosa* YL-1	YPD medium	Glucose: 50 Yeast extract: 10	15.1	Immunomodulatory activity	[62]
*R. mucilaginosa* GUMS16	YPG medium	Peptone: 20 Glycerol: 25	28.5	Antioxidation and high molecular weight	[63]
*R. mucilaginosa* YMM19	PDB medium	Glucose: 20	9.74	Emulsifying	[64]

The impact of BPE media on EPS production was determined by estimating the yield per gram of dry biomass. The EPS yield in YM broth was 76.3 ± 19.44 mg/g dry biomass. On the other hand, EPS yields obtained in BPE media were 160.1 ± 22.09, 115.9 ± 18.72, 134 ± 22.35, 115 ± 19.14 mg/g dry biomass at 50, 100, 150, and 200 g/L, respectively. Our results demonstrated that BPE media enhanced EPS production in *R. mucilaginosa* UANL-001L by 50 to 100% compared to YM broth. As we previously mentioned, BPE contains sugars such as starch and free sugars. It has been reported that sugars like sucrose increase up to 50% of the EPS production [65]. Our results suggest that *R. mucilaginosa* UANL-001L used sugars in the banana peel to produce EPS, which correlates with previous reports [36]. Oleaginous yeasts (e.g., genera *Rhodotorula*, *Yarrowia*, and *Candida*) exhibit advantages for lipid production over other microorganisms (filamentous fungi and microalgae) due to their unicellular form, short duplication times, and their easy cultivation in large reactors. Interestingly, they have the ability to grow on a diversity of carbon sources, including low-cost fermentation media such as agro-industrial wastes [66, 67].

The total lipid content in *R. mucilaginosa* UANL 001L at 50, 100, 150, and 200 g/L BPE was 1.3 ± 0.14, 1.8 ± 0.19, 1.8 ± 0.21, and 2.1 ± 0.26 g/L, while 2.4 ± 0.24 g/L were observed in YM broth (Fig. 2a). Lipid production (g/L) in different oleaginous yeast (*Cryptococcus corvatus*, *Rhodotorula glutinis*, and *Yarrowia lipolytica*) has ranged from 1 to 3 g/L, which is in agreement with our results [52, 75, 76]. Regarding species of the genus *Rhodotorula*, lipid concentration has been reported between 1.5 and 3 g/L. For *R. glutinis*, lipid production was 3 g/L [77], while using cassava wastewater, maximum production of 1.71 g/L was reached [78]. In the case of strains from *R. mucilaginosa*, lipid content reached a concentration of 1.76 g/L when dried durian peel was used as a substrate. Interestingly, biomass content was 11.02 g/L obtaining a lipid concentration of 16% [71].

The ability to produce and store lipids (e.g., triacylglycerol) influences the lipid content [76]. Our results demonstrated a value of 455 ± 25.17 mg of lipid content (45.5% dry biomass) in YM broth. In comparison, the maximum yield in BPE media was reached at 150 g/L 553 ± 10.69 mg/g of dry biomass (55.3% dry biomass) and 200 g/L (539 ± 21.06 mg/g of dry biomass (53.9% dry biomass) (Fig. 2b).

Similar results have also been reported in earlier studies. It has been reported that some oil-producing microorganisms, such as microalgae, had a lipid content of 49.82 and 52.5% in *Chlorella vulgaris* species, while 46.81% was reported in *Scenedesmus dimorphus* [79, 80]. Moreover, Karatay [20] showed that lipid accumulation could be optimized from 38.5 to 69.5% by modifying sugar and ammonium sulfate concentrations in *R. mucilaginosa*. Oleaginous yeast can achieve a maximum lipid accumulation after 5–7, compared to microalgae genres like *Chlorella*, *Chlamydomonas*, and *Desmodesmus*, which take 8–12 days [81–83]. The lipid yields in different strains of *R. mucilaginosa* are summarized in Table 5. Maximum lipid content when wheat bran hydrolysate was used without adding peptone or a nitrogen source was 370 mg/g [72]. The carbon and nitrogen sources (pure glycerol and yeast extract) and the C/N ratio were demonstrated to be significant factors for lipid accumulation in *Rhodotorula mucilaginosa* LP-2 [73]. Moreover, a mutated strain of *Rhodotorula mucilaginosa* was able to grow in seawater. It produced a lipid content of 65 ± 5% as compared to the pure water (primary nutrient source) [21].

**Table 5:** Lipids yield per dry biomass in different *R. mucilaginosa* strains

Microorganism	Substrate^a^	Substrate concentration (g/L)	Yield lipids (mg/g)	Additional details	References
*R. mucilaginosa* UANL001	Banana peel extract	50	483 ± 20		This study
		100	511 ± 21		
		150	553 ± 10		
		200	538 ± 21		
*R. mucilaginosa*	Sea water + glycerol	Glycerol: 60	650	Mutated strain for high lipid content	[21]
*R. mucilaginosa* ATCC 66034	YPD + potato wastewater + glycerol	Glycerol: 30	95 ± 11	Cultured at 20 °C	[54]
*R. mucilaginosa* ATCC 66034	Glycerol	50	102	Peptone 20 g/L and yeast extract 10 g/L	[58]
*R. mucilaginosa KKUSY14*	Durian peel	1000 g	162	Hydrolysate resuspended in water	[71]
*R. mucilaginosa Y-MG1*	Wheat bran	Reducing sugars: 60	370	Acid hydrosilated in 4 L reactor	[72]
*R. mucilaginosa* LP-2	Crude glycerol + yeast extract	Glycerol: 50 Yeast extract: 5	600	C/N ratio of 65	[73]
*R. mucilaginosa* LP-2	Peptone + malt extract	2.036 + 1.515	250		[74]

*YPD* yeast extract–peptone–dextrose medium

^a^Main carbon source

### Fatty acids profile

Finally, the effect of BPE on the fatty acids profile of *R. mucilaginosa* UANL 001L was evaluated. The characterization of the produced fatty acids indicated six major compounds with differences in carbon chain length and saturation: myristic acid, palmitic acid, stearic acid, methyl oleate, ricinoleic acid, and nonadecanoic acid. Table 6 shows the profile of fatty acids identified in *R. mucilaginosa* UANL 001 L in YM broth and BPE. Palmitic (16:0) and stearic acids (18:0) were the most abundant in BPE and YM broth. These fatty acids have been previously reported to be the most common in *Rhodotorula* species and other oleaginous yeasts [75, 84, 85]. Interestingly, an increase of 14.2% in oleic acid (18:1) production was observed when *R. mucilaginosa* UANL 001 grew in 100 g/L BPE compared to YM broth. Oleic acid is an intermediate for detergents, plasticizers for duplicating inks, stabilizers, and emulsifiers, among others [86]. Besides, in biodiesel production, the high content of unsaturated fatty acids methyl esters (FAME) like methyl oleate (MO) is desired to improve ignition quality and fuel stability [84, 87]. When the yeast was cultured in BPE, ricinoleic acid (18:1) was also increased (5.5%). This molecule has attracted growing interest in the chemical industry due to its reactivity and capacity for creating several derivatives products like paints, polymers, fuel feedstock, lubricants, and foams [88].

**Table 6:** Profile of fatty acids in *Rhodotorula mucilaginosa* UANL 001L in YM broth and BPE 100 g/L

Fatty acid	BPE 100 g/L	Yeast-Malt
Myristic acid (14:0)	5.6%	6.4%
Palmitic acid (16:0)	34.7%	40.8%
Stearic acid (18:0)	28.2%	41.7%
Oleic acid (18:1)	21%	6.8%
Ricinoleic acid (18:1)	6.5%	< 1%
Nonadecanoic acid (19:0)	4%	< 5%

## Conclusion

This study demonstrates that banana peel is a potential cheap substrate for producing carotenoids, EPS, and fatty acids using *Rhodotorula mucilaginosa* UANL-001L. The use of this substrate resulted in increased biosynthesis of some valuable products in *Rhodotorula mucilaginosa* UANL-001L compared with that in the YM control medium. Interestingly, the use of BPE promoted an increase in the bioproduction of EPS. For the case of carotenoid production, our results have direct applications for further studying carotenoid production pathways and alternative metabolic flux pathways in the *Rhodotorula* genre. Although genetic engineering and mutation experiments have already been made in *R. mucilaginosa* with an increase in carotenoid production, there is still work to do at a metabolic level to show the potential of this genre.

Moreover, the predominance of unsaturated fatty acids (e.g., linoleic and ricinoleic acid) in BPE indicated that this growth condition triggers interesting pathways for obtaining oil compounds in *R*. *mucilaginosa* UANL-001L. Furthermore, the production of methyl oleate and ricinoleic acid was significantly increased, which has direct applications in the quality and efficiency of biodiesel production. However, the lack of studies at a gene expression level still makes it difficult to associate these differences with the substrate or the strains. Accordingly, future studies must compare gene expression between *Rhodotorula* species and the effect on different substrates.

Altogether, the results in this work show that the microbial production by *R*. *mucilaginosa* UANL-001L represents a viable option to produce high-value compounds with the potential to be used in more diverse industrial sectors. We demonstrated the feasibility of using agro-industrial waste as a promising, cheap, and simple-to-obtain medium for producing high-value compounds with industrial applications by *R*. *mucilaginosa* UANL-001L. Therefore, by exploiting different substrates from agro-industrial residues, we can aid in the studies of other non-conventional microorganisms, such as *Rhodotorula*, *Yarrowia*, *Kluyveromyces*, and *Cryptococcus* species, to identify and track changes along the metabolic pathways without genetic alterations while obtaining high-value products of industrial interest. Finally, this process is yet not developed at an industrial scale because of the low biomass yield and the content of individual components. However, the results shown here shine a light on some considerations for improving the process, including genetic engineering, further substrate development (e.g., a combination of waste nutrients sources), and the addition of micro-nutrients.
